# Mortality and Posthospitalization Outcomes in Heart Failure–Focused Chronic Condition Special Needs Plans

**DOI:** 10.1001/jamanetworkopen.2026.5913

**Published:** 2026-04-09

**Authors:** Bassel M. Shanab, David A. Bunn, Yanlei Ma, Nishmi Abeyweera, Andrew S. Oseran, Rishi K. Wadhera, Jessica Phelan, E. John Orav, Jose F. Figueroa

**Affiliations:** 1 Yale University School of Medicine, New Haven, Connecticut; 2 Department of Health Policy and Management, Harvard T.H. Chan School of Public Health, Boston, Massachusetts; 3 Division of General Internal Medicine and Primary Care, Brigham and Women’s Hospital, Boston, Massachusetts; 4 Harvard Business School, Boston, Massachusetts; 5 Harvard Medical School, Boston, Massachusetts; 6 Division of Cardiology, Beth Israel Deaconess Medical Center, Boston, Massachusetts

## Abstract

This cross-sectional study examines the association of Chronic Condition Special Needs Plans vs conventional Medicare Advantage plans with quality of care and outcomes after hospitalization in patients with heart failure.

## Introduction

Insurance coverage for older adults with heart failure (HF) is rapidly shifting and becoming more complex with the growth of Medicare Advantage (MA).^1^ Within MA, some patients with HF enroll in Chronic Condition Special Needs Plans (C-SNPs), which are specialized plans for people with chronic conditions, including HF.^2^ Medicare Advantage insurers market C-SNPs as being beneficial for patients with chronic conditions by offering enhanced specialist access, tailored formularies, and improved care coordination. Although 86% of C-SNPs are designed specifically for patients with HF and have been growing rapidly,^3^ evidence remains limited on the association of HF C-SNPs with quality of care or outcomes after hospitalization compared with conventional MA plans.

## Methods

This cross-sectional study used 100% Medicare encounter data to identify MA beneficiaries hospitalized with a primary diagnosis of HF between January 1, 2021, and September 30, 2022, using HF-specific *International Statistical Classification of Diseases, Tenth Revision* codes. The Harvard Institutional Review Board approved this study and granted a waiver of informed consent because of the use of deidentified administrative data. The study followed the STROBE reporting guideline.

The primary outcome was mortality at 30 and 90 days from the index hospitalization date. Secondary outcomes included readmission rate and hospital revisit rate, including inpatient stays, observation stays, and emergency department visits. Mortality was measured from date of admission. Readmissions and hospital revisits were measured from date of discharge.

Risk-adjusted outcomes were compared between patients enrolled in HF C-SNPs vs conventional MA plans offered in the same counties using logistic regression models with winsorized inverse probability weighting (IPW) of MA beneficiaries. Weighting included variables for age, sex, self-reported race and ethnicity (Black, Hispanic, White, other [American Indian or Alaska Native, Asian, Native Hawaiian or Pacific Islander, unknown; aggregated due to small sample numbers]), dual-eligibility status, census region, and rurality. Race and ethnicity variables were included given baseline MA enrollment differences compared with White beneficiaries. Beneficiary-level models were then performed, accounting for demographics, Elixhauser comorbidities, state fixed effects, and hospital random effects, along with robust SEs. For interpretability, model results are presented as rate differences, calculated as marginal effects. Results were considered significant if the 95% CI did not include 0. Analyses were performed between March 24, 2025, and February 3, 2026, using Stata, version 19 (StataCorp LLC). Additional methodological details are provided in the eMethods in Supplement 1.

## Results

The sample included 198 210 patients hospitalized for HF, of whom 6.8% were enrolled in C-SNPs (55.4% aged <65 to 74 years and 44.6% aged 75 to >85 years; 51.7% female and 48.3% male) and 93.2% in conventional MA plans (42.5% aged <65 to 74 years and 57.4% aged 75 to >85 years; 49.6% female and 50.4% male). After IPW, the beneficiary characteristics were well balanced between groups (Table). After adjustment, mortality was similar, but not significant among patients in HF C-SNPs vs conventional MA plans (30-day mortality, −0.28 [95% CI, −1.13 to 0.57] percentage points; 90-day mortality, 0.40 [95% CI, −0.78 to 1.57] percentage points) (Figure). No significant differences were found in readmission rates (30-day readmissions, −0.66 [95% CI, −1.79 to 0.48] percentage points; 90-day readmission rates, 0.98 [95% CI, −0.40 to 2.36] percentage points) or hospital revisit rates (30-day revisits, −0.73 [95% CI, −2.04 to 0.58] percentage points; 90-day revisits, 1.07 [95% CI, −0.40 to 2.55] percentage points).

**Table.** Characteristics of Hospitalized Patients With HF in C-SNPs vs Conventional MA Plans

Characteristic	Patients, unweighted No. (%)		SMD^b^	Propensity score–weighted percentage (95% CI)^c^		SMD^b^
	C-SNPs	Conventional MA plans^a^		C-SNPs	Conventional MA plans^a^	
No. of patients	13 478	184 732	NA	NA	NA	NA
Age group, y						
<65	2508 (18.6)	20 523 (11.1)	0.21	13.0 (12.3-13.8)	11.6 (11.5-11.8)	0.06
65-74	4959 (36.8)	58 070 (31.4)	0.11	34.0 (32.9-35.2)	31.8 (31.6-32.0)	0.07
75-84	4238 (31.4)	63 232 (34.2)	0.06	33.2 (32.0-34.4)	34.0 (33.8-34.4)	0.03
≥85	1773 (13.2)	42 907 (23.2)	0.26	19.7 (18.7-20.8)	22.5 (22.4-22.7)	0.10
Sex						
Female	6974 (51.7)	91 633 (49.6)	0.04	48.2 (47.0-49.5)	49.7 (49.5-49.9)	0.04
Male	6504 (48.3)	93 099 (50.4)	0.04	51.8 (50.4-53.0)	50.3 (50.1-50.5)	0.04
Race and ethnicity						
Hispanic	1915 (14.2)	20 180 (10.9)	0.10	12.9 (12.1-13.7)	11.2 (11.0-11.3)	0.08
Non-Hispanic Black	4206 (31.2)	35 055 (19.0)	0.28	20.2 (19.3-21.2)	19.8 (19.6-19.9)	0.02
Non-Hispanic White	7103 (52.7)	122 466 (66.3)	0.28	63.2 (62.0-64.4)	65.4 (65.2-65.6)	0.07
Other^d^	254 (1.9)	7031 (3.8)	0.12	3.7 (3.2-4.3)	3.7 (3.6-3.8)	0.003
Medicaid dual eligibility	4901 (36.4)	34 682 (18.8)	0.40	18.4 (17.6-19.1)	19.9 (19.7-20.1)	0.05
Original Medicare eligibility						
Old age or survivor insurance	7016 (52.1)	129 563 (70.1)	0.38	66.2 (65.1-67.3)	68.9 (68.7-69.1)	0.08
Disability and/or ESKD	6462 (47.9)	55 169 (29.9)	0.38	33.8 (32.7-34.9)	31.1 (30.9-31.3)	0.08
Region						
Midwest	1287 (9.5)	48 292 (26.1)	0.22	23.3 (22.1-24.5)	25.0 (24.8-25.2)	0.06
Northeast	131 (1.0)	8265 (4.5)	0.44	4.3 (3.6-5.0)	4.2 (4.1-4.3)	0.004
South	11 269 (83.6)	85 215 (46.1)	0.85	51.2 (49.9-52.5)	48.7 (48.4-48.9)	0.07
West	791 (5.9)	42 960 (23.3)	0.51	21.2 (19.9-22.5)	22.1 (21.9-22.3)	0.03
Rural	3480 (25.8)	13 176 (7.1)	0.52	8.5 (8.0-8.9)	8.4 (8.2-8.5)	0.01
Comorbidities, mean (SD)	4.1 (1.8)	3.9 (1.9)	0.09	4.0 (4.0-4.1)	3.9 (3.9-3.9)	0.07

Abbreviations: C-SNP, Chronic Condition Special Needs Plan (specifically for patients with heart failure); ESKD, end-stage kidney disease; HF, heart failure; MA, Medicare Advantage; NA, not applicable; SMD, standardized mean difference.

^a^ Conventional MA plans are those that are either health maintenance organization, point-of-service, or preferred provider organization plans.

^b^ The SMD is considered negligible when less than 0.10.

^c^ Inverse probability weighting, winsorized at the 95th percentile, was applied for the weighted comparison. Propensity scores were estimated using a logistic regression model in which the dependent variable was a binary indicator for HF C-SNP enrollment, and the explanatory variables included age, sex, race and ethnicity, Medicaid dual eligibility, US region, and rurality. The CIs reflect robust SEs.

^d^ Included American Indian or Alaska Native, Asian, Native Hawaiian or Pacific Islander, or unknown and were aggregated due to small cell sizes.

**Figure.**
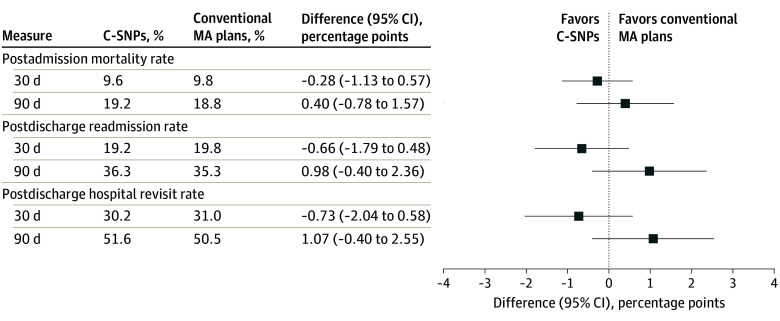
Forest Plot of Adjusted Differences in Mortality and Posthospitalization Outcomes Between Chronic Condition Special Needs Plans (C-SNPs) and Conventional Medicare Advantage (MA) Plans Adjusted rates and rate differences were calculated as marginal means from a logistic regression model. Inverse probability–weighted logistic regression models were estimated for each outcome, with weights winsorized at the 95th percentile. The dependent variable was the given outcome, and the key explanatory variable was a binary indicator of heart failure C-SNP enrollment. Models were adjusted for age, sex, race and ethnicity, Medicaid dual eligibility, Elixhauser comorbidities, state fixed effects, and hospital random effects, along with robust SEs. Mortality was measured from date of admission. The hospital revisit rate was defined as a beneficiary having at least 1 of the following within the designated period measured from date of discharge: an inpatient stay, an observation stay, or an emergency department visit.

## Discussion

This cross-sectional study found that enrollment in C-SNPs was not associated with substantial improvements in mortality or hospital revisit rates among hospitalized patients with HF compared with conventional MA plans. These findings suggest that HF C-SNPs may not offer the value promised for hospitalized patients.

This study had some limitations. Although we addressed potential selection bias by IPW and controlling for beneficiary characteristics, residual confounding was possible. Additionally, the findings are generalizable only to hospitalized patients with HF, and the analyses used administrative data, which lack clinical detail.

Our study also has important policy implications. The federal government has debated termination of C-SNPs to simplify plan choice, reduce administrative MA complexity, and help alleviate potential detrimental plan selection. However, MA insurers have argued successfully to retain them due in part to attractive plan benefits that may improve care for beneficiaries with chronic conditions.^2,4,5^ There is still substantial concern that these supplemental benefits are not well used or easily accessible, partially due to limited awareness, and therefore may function more as an insurer marketing strategy than as a true benefit.^6^ Moving forward, policymakers should reconsider C-SNPs’ special designation until evidence shows their value over existing plans, especially given higher enrollment among historically marginalized populations.

## Notes

Supplement 1.
eMethods.

eReferences.

Supplement 2.
Data Sharing Statement
