# Supplementary material for: Mortality and Posthospitalization Outcomes in Heart Failure–Focused Chronic Condition Special Needs Plans
Source: JAMA Netw Open. 2026 Apr 9;9(4):e265913. doi: 10.1001/jamanetworkopen.2026.5913 (PMC13067005; doi:10.1001/jamanetworkopen.2026.5913)
Supplement: Supplement 1. — eMethods. eReferences. [file jamanetwopen-e265913-s001.pdf]

Supplemental Online Content

Shanab BM, Bunn DA, Ma Y, et al. Chronic condition special needs plan outcomes in patients with heart failure. *JAMA Netw Open*. 2026;9(4):265913
doi:10.1001/jamanetworkopen.2026.5913

eMethods

eReferences

This supplemental material has been provided by the authors to give readers additional information about their work.

© 2026 Shanab BM et al. *JAMA Network Open*.

eMethods

Further Details on Data & Study Sample

We utilized the Centers for Medicare & Medicaid Services (CMS) Chronic Condition Data Warehouse for heart failure (HF)-specific International Classification of Diseases (ICD)-10 codes.[1] In addition, we utilized the Research Triangle Institute (RTI) race code to identify beneficiaries' race and ethnicity.[2] The RTI race code is determined by applying an algorithm to Medicare beneficiary self-reported race and ethnicity data to improve classification accuracy. Beneficiaries were limited to those residing in counties only where cardiovascular disease or HF-specific Chronic Condition-Special Needs Plans (C-SNPs) were offered.

Additional Details about the Study Outcomes and Statistical Analysis

Mortality was defined as any death occurring within 30-days or 90-days from the date of the index admission date of hospitalization. Hospital readmission was defined as a beneficiary having an inpatient admission within 30-days or 90-days from date of the hospital discharge. A hospital revisit was defined as a beneficiary having at least one of the following events: an inpatient hospitalization stay, an observation stay, or an emergency department visit within the 30-days or 90-days from the date of the hospital discharge. If they had none of these events, then they were not found to have a hospital revisit. The reason we evaluated for the hospital revisits is because prior data has shown that Medicare Advantage (MA) plans are increasingly lowering inpatient admission stays in favor of observation stays or emergency department treat-and-release stays.[4]

© 2026 Shanab BM et al. *JAMA Network Open*.

Next, risk-adjusted outcomes were compared between hospitalized patients enrolled in HF C-SNPs versus conventional MA plans offered in the same counties, using logistic regression models with inverse probability weighting of MA beneficiaries, which included variables for age, sex, race/ethnicity (using RTI race code which applies an algorithm on self-reported data), dual-eligibility status, regions, and rurality. To address additional variability potentially introduced via inverse probability score weighting, we winsorized the weights at the 95th percentile, as well as using robust standardized errors via a sandwich variance estimator.[3] Beneficiary-level models were then performed, accounting for demographics, Elixhauser-comorbidities, state fixed-effects, and hospital random-effects.

eReferences

1. Centers for Medicare & Medicaid Services. Chronic conditions. Chronic Conditions Data Warehouse. Accessed October 6, 2025. https://www2.ccwdata.org/condition-categories-chronic
2. Research Data Assistance Center. Research Triangle Institute (RTI) Race Code. Available from: https://resdac.org/cms-data/variables/research-triangle-institute-rti-race-code
3. Ma, Y., Giffin, A., He, J., & Lee, H. (2025). Demystifying stabilization in inverse probability of treatment weighting. Journal of Biopharmaceutical Statistics, 1–15. https://doi.org/10.1080/10543406.2025.2604126
4. Beckman AL, Frakt AB, Duggan C, et al. Evaluation of Potentially Avoidable Acute Care Utilization Among Patients Insured by Medicare Advantage vs Traditional Medicare. *JAMA Health Forum*. 2023;4(2):e225530. Published 2023 Feb 3. doi:10.1001/jamahealthforum.2022.5530

© 2026 Shanab BM et al. *JAMA Network Open*.
