# Supplementary material for: Mortality and Posthospitalization Outcomes in Heart Failure–Focused Chronic Condition Special Needs Plans
Source: JAMA Netw Open. 2026 Apr 9;9(4):e265913. doi: 10.1001/jamanetworkopen.2026.5913 (PMC13067005; doi:10.1001/jamanetworkopen.2026.5913)
Supplement: Supplement 2. — Data Sharing Statement [file jamanetwopen-e265913-s002.pdf]

Data Sharing Statement

Data
Data available: No

Additional Information
Explanation for why data not available: DUA restricts our ability to share data but anyone can apply for same data from CMS directly.
